# Bridging the Gap Between Pharmacogenomic Discovery and Clinical Implementation: Insights from Selected Studies on Inter-Individual Variability in Drug Response

**DOI:** 10.3390/jpm16040221

**Published:** 2026-04-17

**Authors:** Su-Jun Lee

**Affiliations:** Department of Pharmacology and Pharmacogenomics Research Center, Inje University College of Medicine, Inje University, Busan 47392, Republic of Korea; 2sujun@inje.ac.kr; Tel.: +82-518905911

## 1. Introduction

Inter-individual variability in drug efficacy and toxicity remains a major challenge in modern healthcare, particularly as aging populations are increasingly exposed to polypharmacy and complex treatment regimens [1,2]. Pharmacogenomics has emerged as a central component of precision medicine because it provides a framework with which to explain how genetic variation in drug-metabolizing enzymes, transporters, receptors, and related pathways contributes to differences in therapeutic response and adverse drug reactions [1,2]. Despite substantial progress in genomic discovery, the routine clinical application of pharmacogenomics remains limited, and many treatment decisions are still based on empirical prescribing rather than genetically informed strategies [1,3]. This gap reflects not only incomplete evidence for some drug–gene pairs but also broader implementation barriers, including the need for standardized interpretation, clinical decision support, provider education, reimbursement, and equitable access to testing [1,3,4]. Over the past decade, the field has advanced from candidate-gene studies and reactive single-gene testing toward more structured models supported by CIPIC and DPWG guidelines, which have facilitated the translation of genotype results into therapeutic recommendations [2,4,5]. More recently, attention has shifted toward preemptive multigene panel testing and health-system integration, highlighting the importance of embedding pharmacogenomic information into routine clinical workflows before prescribing decisions are made [2,3]. In this context, this Special Issue addresses the persistent gap between pharmacogenomic discovery and clinical implementation by bringing together studies that examine both the biological basis of inter-individual variability and the practical challenges of applying this knowledge in healthcare settings.

## 2. Recent Studies That Apply Pharmacogenomics Data to Clinical Patients

Recent advances in pharmacogenomics (PGx) have demonstrated that genomically guided prescribing is increasingly transitioning from a theoretical framework to a clinically actionable strategy across diverse healthcare settings. In dermatology, recent evidence has shown that polymorphisms in SLC19A1 and COL18A1 are associated with methotrexate treatment failure in psoriasis patients, highlighting the clinical utility of pharmacogenomic markers in predicting therapeutic outcomes and guiding treatment transitions (contribution 1). The PREPARE study provided compelling multicenter evidence that preemptive panel-based PGx testing significantly reduces adverse drug reactions in real-world clinical practice, establishing a benchmark for large-scale implementation [6]. Extending these findings into psychiatry, Skokou et al. [7] showed that PGx-guided treatment of patients with major psychiatric disorders reduced adverse events, hospitalizations, and healthcare costs, highlighting genomics’ potential to improve outcomes in complex, trial-and-error therapeutic areas. In addition, whole-genome sequencing of case–parent trios among patients with orofacial clefts has revealed clinically relevant variants in multiple pharmacogenes, including CYP enzymes and drug transporters, demonstrating how rare and population-specific genetic variation can influence drug metabolism and therapeutic responses in real-world patient populations (contribution 2). At the institutional level, it was demonstrated that pharmacogenomic data can be successfully integrated into electronic health records and linked to automated clinical decision support systems, thereby enabling real-time genotype-guided prescribing within routine workflows [8]. In regard to cardiovascular medicine, it was reported that CYP2C19-guided antiplatelet therapy after percutaneous coronary intervention is feasible across multiple centers and can inform treatment selection for high-risk populations [9]. Furthermore, it was demonstrated that genotype-guided antiplatelet strategies can be effectively applied to neuro-interventional procedures, including intracranial aneurysm repair [10]. In urology, pharmacogenomic variation in CYP3A4, CYP3A5, and UGT2B7 has been shown to significantly influence silodosin exposure and therapeutic responses in patients with benign prostatic hyperplasia, illustrating how multi-gene interactions can modulate both pharmacokinetics and clinical efficacy in routine care (contribution 3). Importantly, it was reported that PGx implementation is expanding across diverse healthcare systems, with high rates of actionable variants and positive patient perceptions observed in outpatient settings in Asia, underscoring the global relevance of personalized prescribing [11]. Similarly, Tremmel et al. [12] described an integrated hospital-based PGx workflow that combines pharmacogenomic and drug–drug interaction data to support medication management across inpatient and outpatient care, reflecting a more mature and clinically embedded implementation model. In the acute-care setting, McDermott et al. [13] highlighted the potential utility of PGx testing during hospital admission, particularly for older patients with multimorbidity, although further interventional studies are needed to confirm its impact on outcomes. It was demonstrated that PGx-guided approaches are highly relevant in hyper-polypharmacy populations, identifying clinically actionable drug–gene interactions in nearly half of patients and supporting the role of PGx in complex medication optimization [14]. Taken together, these studies illustrate that PGx implementation is now being actively evaluated across cardiology, psychiatry, neurology, primary care, acute hospital medicine, and polypharmacy management, with emerging evidence of improved prescribing decisions and reduced adverse drug responses. However, they also reveal that the major remaining challenges are no longer analytical but instead operational, including integration into clinical workflows, interoperability of electronic systems, reimbursement structures, and clinician readiness to apply genomic information in practice. Accordingly, the next phase of personalized pharmacotherapy will require not only continued accumulation of clinical evidence but also the parallel development of robust clinical decision support tools, scalable implementation models, and innovative educational programs for physicians and pharmacists to fully realize the potential of pharmacogenomics in routine care.

## 3. Clinical Decision Support Tools, Software, and Models

The integration of pharmacogenomics into clinical practice has been accelerated by the development of clinical decision support (CDS) tools, software platforms, and predictive models that translate genomic data into actionable prescribing guidance. Early foundational work by Bell et al. [15] demonstrated that pharmacogenomic results could be embedded within electronic health records (EHRs) and linked to automated alerts, enabling clinicians to receive genotype-guided recommendations at the point of care. This study established the feasibility of real-time genomic decision support and highlighted the importance of workflow integration for successful implementation. Similarly, they noted that a comprehensive institutional framework enables preemptively generated pharmacogenomic data to be converted into clinically interpretable phenotypes and incorporated into both inpatient and outpatient clinical decision support systems [16]. Their work underscored that effective implementation requires not only data generation but also robust knowledge translation and governance infrastructure. Subsequent studies provided evidence that CDS systems can directly influence prescribing behavior. It was demonstrated that clinicians altered medication choices in response to pharmacogenomic alerts, avoiding high-risk drugs when genotype-based recommendations were presented, thereby providing critical validation that clinical decision support tools can bridge the gap between genomic information and clinical action [17]. In parallel, it was reported that advances in interoperability have been achieved through the development of a standards-based clinical decision support framework using Fast Healthcare Interoperability Resources (FHIRs) and CDS Hooks [18]. This approach enabled pharmacogenomic decision support to be deployed across different health IT systems, representing a key step toward scalable and portable implementation.

More recent efforts have focused on expanding pharmacogenomic CDS into broader clinical settings. Sharma et al. [19] developed a primary-care-oriented pharmacogenomic CDS system that emphasized usability, standardized result presentation, and integration into routine prescribing workflows. Their study is particularly important, as it extends precision prescribing beyond specialized centers into primary care, where most chronic disease management and polypharmacy occur. In addition, it was reported that large-scale implementation of preemptive pharmacogenomic testing linked to automated clinical decision support systems has been achieved within a major academic health system [8]. Their program included dozens of gene–drug pairs, hundreds of prescribing alerts, and testing prompts, illustrating how pharmacogenomics can be operationalized at the institutional scale. Collectively, these studies demonstrate that CDS tools are central to translating pharmacogenomic knowledge into clinical practice. By reducing the cognitive burden on clinicians and providing real-time, evidence-based recommendations, CDS systems enable more precise, safe, and effective medication use. Furthermore, the evolution from single-institution implementations to interoperable, standards-based platforms reflects a broader shift toward scalable precision medicine infrastructure. Importantly, these tools also facilitate the integration of complex genomic data into routine care without requiring clinicians to have advanced expertise in genomics, thereby democratizing access to personalized prescribing.

## 4. Innovative Medical Education Programs for Physicians and Pharmacists

Alongside technological advancements, innovative educational programs for physicians and pharmacists have emerged as a critical component in the implementation of pharmacogenomics. Formea et al. [20] developed a case-based pharmacogenomics education program for pharmacists, demonstrating significant improvements in knowledge and competency following structured training. This work highlighted the importance of applied, patient-centered learning approaches, which enable clinicians to translate theoretical genomic concepts into practical clinical decision-making. It was further demonstrated that continuing-education programs in pharmacogenomics improve pharmacists’ readiness to incorporate genomic information into patient care, thereby emphasizing the value of ongoing professional training [21]. These findings indicate that ongoing professional development is essential for preparing the current workforce for precision medicine. Building on these foundations, it was demonstrated that a pharmacogenomics education program can be implemented across a multicampus health system, creating a scalable model for disseminating genomic knowledge in diverse clinical environments [22]. Their program demonstrated that structured educational frameworks can overcome institutional variability and resource limitations, thereby supporting widespread adoption. Crown et al. [23] expanded this approach by designing a comprehensive continuing professional development (CPD) program that combined online learning, workshops, and case-based exercises. Importantly, this study showed that education not only improves knowledge and confidence but also leads to actual changes in clinical practice, with participants applying pharmacogenomic principles in real patient care settings. Interprofessional education has also emerged as a key strategy. It was demonstrated that team-based learning models involving both medical and pharmacy students improve interprofessional collaboration and pharmacogenomics competency [24]. These findings suggest that collaborative training environments can enhance our understanding of roles and responsibilities in precision medicine. Similarly, it was demonstrated that interprofessional educational experiences focused on defining the roles of physicians and pharmacists can support effective pharmacogenomic implementation, emphasizing that successful precision medicine requires coordinated efforts across healthcare disciplines [25]. Recent studies have also explored scalable educational models. It was demonstrated that both synchronous and asynchronous pharmacogenomics training programs can effectively improve knowledge and confidence among pharmacists [26]. This finding is particularly important in the context of global implementation, as it supports the feasibility of remote and flexible education formats. Overall, these educational initiatives demonstrate that equipping healthcare professionals with the necessary knowledge, skills, and confidence is essential for the successful integration of pharmacogenomics into clinical practice.

## 5. Significance of Clinical Decision Support Tools and Medical Education

The successful implementation of personalized drug therapy depends on integration achieved through the synergy of technology and education. Importantly, patient-centered factors have emerged as critical determinants of successful pharmacogenomic implementation, with studies in primary care showing that underserved populations strongly favor pre-emptive testing while emphasizing the need for clear communication, accessible result interpretation, and clinician engagement (contribution 4). Clinical decision support tools enable the translation of complex genomic data into actionable prescribing recommendations, thereby reducing variability in drug responses and improving patient safety. At the same time, educational programs ensure that healthcare professionals are well-equipped to interpret and apply these recommendations appropriately. Without adequate training, even the most advanced CDS systems may fail to achieve their intended impact. Furthermore, interprofessional collaboration between physicians and pharmacists enhances the effective use of pharmacogenomic information in clinical decision-making. As healthcare systems increasingly face challenges related to aging populations and polypharmacy, these combined efforts are becoming even more critical. The integration of CDS and education represents a foundational strategy for advancing precision medicine and achieving more individualized, effective, and safe pharmacotherapy. Ultimately, these approaches contribute to the transformation of healthcare from a one-size-fits-all model into a data-driven, patient-centered paradigm.

## 6. Future Directions

Future efforts should focus on developing interoperable, scalable pharmacogenomic CDS systems that can be seamlessly integrated across diverse healthcare settings, including primary care and community pharmacies. As illustrated by studies on vitamin D pharmacogenomics, future precision medicine will require integrated decision-making that incorporates multi-gene genetic variants, their functional consequences, and additional factors such as environmental and nutritional influences, moving beyond single-gene approaches toward more comprehensive, system-level strategies (contribution 5). In parallel, educational programs should evolve toward long-term, case-based, and interprofessional models embedded within clinical training and practice. The convergence of real-world data, artificial intelligence, and adaptive healthcare systems will further amplify the impact of pharmacogenomics on clinical outcomes. Ultimately, an integrated approach that combines technological innovation with personnel development will be essential for fully realizing the potential of personalized medicine.
